# Histiocytic Glomerulopathy With Noncrystalline Inclusion Associated With IgG-Kappa Plasma Cell Dyscrasia

**DOI:** 10.1016/j.xkme.2023.100617

**Published:** 2023-02-15

**Authors:** Ai Katsuma, Masahiro Okabe, Hiroyuki Ueda, Takashi Ehara, Yutaka Yamaguchi, Yoichi Miyazaki, Takashi Yokoo

**Affiliations:** 1 Division of Nephrology and Hypertension, Department of Internal Medicine, Jikei University School of Medicine, Tokyo, Japan; 2 Department of Histopathology, Shinshu University School of Medicine, Matsumoto, Japan; 3 Yamaguchi's Pathology Laboratory, Chiba, Japan

**Keywords:** Crystal-storing histiocytosis, histiocytic glomerulopathy, immunoelectron microscopy, lysosome indigestion/constipation, monoclonal gammopathy with renal significance (MGRS), noncrystalline storing histiocytosis, plasma cell dyscrasia

## Abstract

The kidney pathology of monoclonal gammopathy of renal significance varies greatly. In this report, we present a woman in her 20s with nephrotic syndrome and monoclonal immunoglobulin G kappa (serum and urine) without diabetes. She had a family history of nephrotic syndrome as well as hematologic and connective tissue disorders. A kidney biopsy showed nodular glomerulosclerosis, with the glomerular capillary full of histiocytes, which were strongly positive for kappa, not lambda. Immunoelectron microscopy revealed that histiocytes had infiltrated the glomerular subendothelial space, and enlarged lysosomes of histiocytes contained kappa light chains, without apparent crystalline formation. Bone marrow examination was negative for malignancy; thus, we diagnosed this case as histiocytic glomerulopathy with noncrystalline inclusion associated with immunoglobulin G-kappa plasma cell dyscrasia. Hematologic treatment with bortezomib and daratumumab decreased her level of serum kappa chain and proteinuria. Two years after diagnosis, her kidney function remained normal, urinary protein level decreased to 1 g/d, and free light-chain ratio decreased to 3.1.

## Introduction

In 2012, the International Kidney Monoclonal Gammopathy Research Group introduced the term monoclonal gammopathy of renal significance (MGRS) to describe hematologic conditions that produce a monoclonal immunoglobulin (Ig) associated with kidney injury. Since then, a variety of kidney diseases have been described in association with MGRS.^1^ We observed a case of IgG-kappa–positive nodular glomerulosclerosis, with numerous histiocytes and without crystal formation. The glomerular basement membrane (GBM) and tubular basement membrane were both kappa negative. Considering that light-chain proximal tubulopathy (LCPT) can be classified into crystalline and noncrystalline subtypes, we thought that this case could be diagnosed as “histiocytic glomerulopathy with non-crystalline inclusion” or “non-crystalline storing histiocytosis.” Although this diagnosis has not been reported in the past, it is considered an important framework for recognizing MGRS.

## Case Report

A Japanese woman in her 20s with no medical history presented to our hospital with edema. She had a unique family history; her maternal grandmother died of nephrotic syndrome in her 50s, and 2 of her maternal grandmother’s sisters died of hematologic disease at the age of 40 years and connective tissue disease at a younger age. Laboratory findings showed nephrotic syndrome (a urinary protein excretion of 9.0 g/d and a serum albumin level of 2.3 g/dL), anemia (hemoglobin level, 9.0 g/dL), and leukopenia (white blood cells, 2,800/μL). Her serum creatinine level was 0.62 mg/dL, with significant microhematuria. There was no evidence of diabetes (hemoglobin A1c level, 6.0%). IgG-kappa monoclonal protein was detected in her serum and urine. The free light-chain ratio was 4.3, and the IgG, IgA, and IgM levels were 719, 48, and 34 mg/dL, respectively, and there was no reduction in the level of complements. The plasma cell percentage was 3% in her bone marrow, and hematologic malignancy was denied by a hematologic work-up.

**Figure 1:**
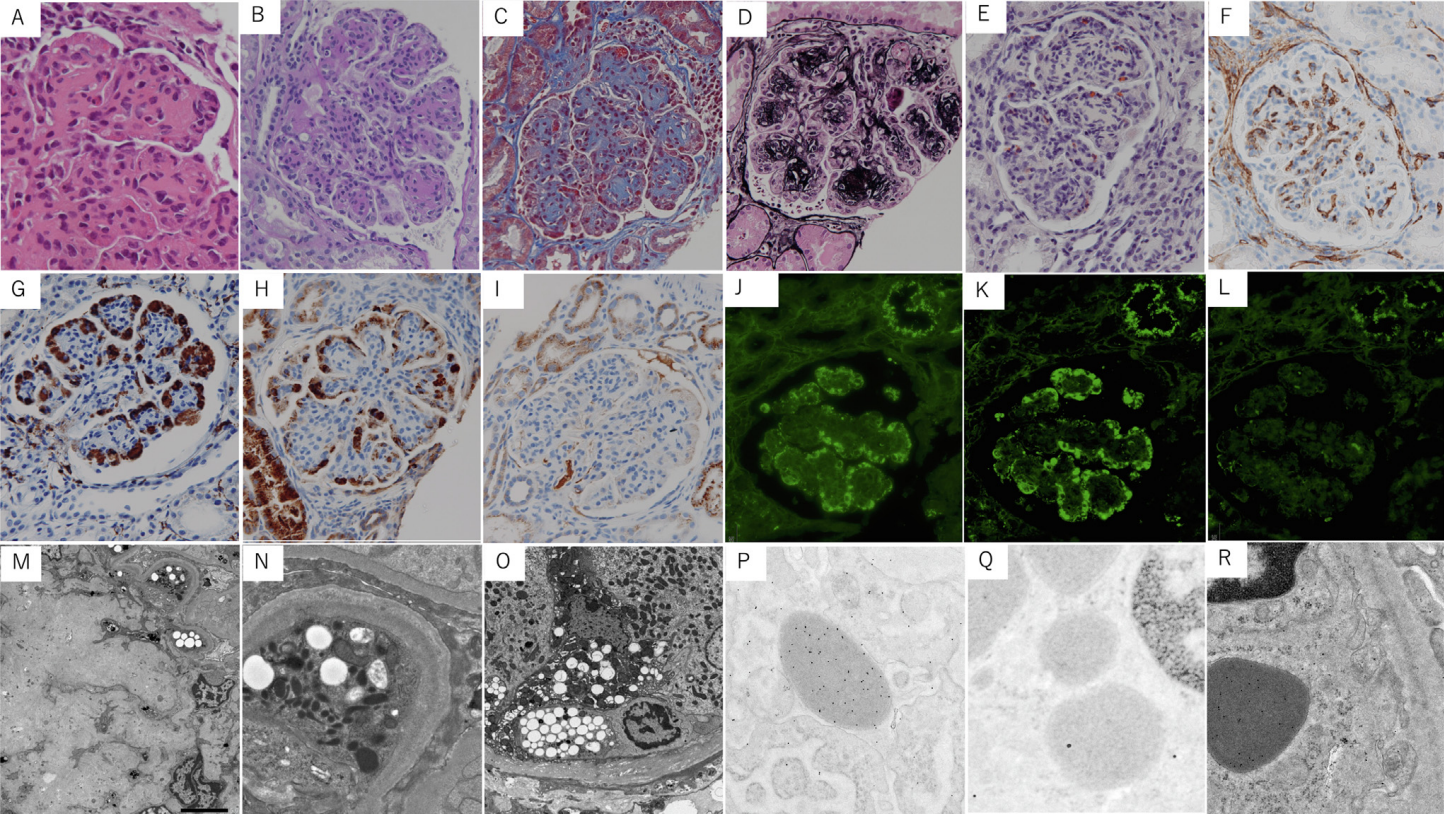
Representative findings of the kidney biopsy. (A-D) The microscopic findings showed nodular glomerulosclerosis with lobulation. The cells infiltrating the glomerular capillary contained fuchsin-positive and periodic acid–Schiff–negative granules. (A) Hematoxylin-eosin stain: original magnification, ×400. (B) Periodic acid–Schiff stain: original magnification, ×400. (C) Masson trichrome stain: original magnification, ×400. (D) Periodic acid–methenamine–silver stain: original magnification, ×400. (E) The mesangial nodules yielded negative results for direct fast scarlet staining, and some eosinophils appeared to be scattered in the glomeruli (Direct fast scarlet stain, Muto Pure Chemicals Co, Ltd: original magnification, ×400). (F) CD34-positive endothelial cells are located in the center of nodular lesions (staining for CD34, Nichirei). (G) The cells infiltrating the glomerular capillaries were CD68 positive (staining for CD68, Dako M0876 PG-M1 clone: original magnification, ×400). (H, I) Immunohistochemical staining of the cytoplasm of capillary histiocytes tested positive for kappa light chains, whereas they tested negative for lambda light chains. (H) Staining for kappa light chains, Nichirei 713891 L1C1 clone: original magnification, ×400. (I) Staining for lambda light chains, Nichirei 713901 HP6054 clone: original magnification, ×400. (J-L) Additional paraffin immunofluorescence staining showed positivity for immunoglobulin (Ig)G and kappa in the histiocytes of glomerular capillaries but negativity for lambda. The glomerular basement membrane and tubular basement membrane were negative for kappa and lambda (immunofluorescence on pronase-digested paraffin sections). (J) IgG, Dako F020202: original magnification, ×400. (K) Kappa, SouthernBiotech 2060-02: original magnification, ×400. (L) Lambda, SouthernBiotech 2070-03: original magnification, ×400. (M-O) Electron microscopy. A large mesangial nodular lesion without any powdery electron-dense deposition. (M) The glomerular subendothelial foam cells (histiocytes) included electron-dense granules and vacuoles. (N) Layered electron-dense deposits were seen in the glomerular basement membrane, inconsistent with monoclonal immunoglobulin deposition disease. Foam cell infiltration was observed below the tubular basement membrane. (O) Few vacuoles were observed in the tubular epithelial cells. (P-R) Immunoelectron microscopy. Immunogold labeling for kappa light chains yielded positive results in lysosomes, whereas they yielded negative results for lambda (P: kappa, Q: lambda, R: kappa; primary antibody for kappa/lambda light chains, Dako; 10-nm gold-conjugated secondary antibody; BBI solutions). (R) Positive kappa signals were not observed along the glomerular basement membrane, a finding inconsistent with monoclonal immunoglobulin deposition disease.

A kidney biopsy revealed a “membranoproliferative glomerulonephritis (MPGN) appearance,” with lobulation and nodular glomerulosclerosis in every glomerulus (Fig 1A-D). We found mild interstitial infiltration of lymphocytes, with mild peritubular capillaritis but no tubulitis. There was no vasculitis or arteriosclerosis. The mesangial nodules yielded negative results for direct fast scarlet staining (Fig 1E). CD34-positive endothelial cells were observed in the center of the nodular lesions, and a cellular infiltrate appeared to be present in the subendothelial space (Fig 1F). Glomerular capillaries were filled with CD68-positive histiocytes, which were positive only for kappa but not lambda following heat-induced antigen retrieval. The nodular lesions were negative for kappa (Fig 1G-I). CD68-positive histiocytes were also observed occasionally in the interstitium (not shown). Immunofluorescence (IF) analysis of frozen tissue showed that C3 was present along the glomerular endocapillary; however, IgG, IgA, IgM, C4, kappa, and lambda were absent. Additional IF staining of paraffin sections tested positive for IgG and kappa in the histiocytes of glomerular capillaries but negative for lambda (Fig 1J-L). The paraffin IF sections were negative for both kappa and lambda in the GBM and tubular basement membrane. Electron microscopy (EM) depicted large nodular mesangial lesions (Fig 1M) as well as spherical electron-dense granules and vacuoles in the cytoplasm of histiocytes in the glomerular capillaries (Fig 1N). These numerous dense granules were assumed to be “lysosomal indigestion/constipation” in the histiocytes.^2^ Focal and segmental electron-dense material was found along with moderate foot process effacement in the GBM. The thickness of the GBM was normal (Fig 1N). In tubular epithelial cells, many dense granules and vacuoles were found, which were also seen in histiocytes (Fig 1O). There was no apparent crystal structure in the histiocytes of the glomeruli or tubulointerstitium. These findings broadly correspond to “MPGN with masked monoclonal immunoglobulin”^3^; however, no typical dense deposits were observed using EM. Light- and heavy-chain deposition disease was also considered based on the positivity of IgG and kappa in the paraffin IF sections; however, the GBM, tubular basement membrane, and vessel wall showed negative findings and the EM findings were not atypical for light- and heavy-chain deposition disease. Paraffin IF analysis of IgG subclasses was not performed. Immunoelectron microscopy was performed to determine the presence of positive signals in the electron-dense granules in the histiocytes and GBM. It revealed that lysosomes were stained with kappa but not lambda (Fig 1P, Q). No signals indicating kappa were present along the GBM or mesangial matrix (Fig 1R). Therefore, we diagnosed this case as “histiocytic glomerulopathy with non-crystalline inclusion associated with IgG-kappa plasma cell dyscrasia.” The patient was treated with bortezomib and dexamethasone (BD) therapy for MGRS because of plasma cell dyscrasia. After 8 courses of BD therapy, her proteinuria decreased (urinary protein excretion, 2 g/d). However, the free light-chain ratio increased from 9 to 32, as did proteinuria (9 g/d) during the 2-month period when BD therapy was suspended because of appendicitis. Thereafter, BD therapy was replaced with daratumumab in combination with BD. Twenty-four months after diagnosis, the urinary protein concentration decreased to 1 g/d, and the free light-chain ratio decreased to 3.1.

## Discussion

We present a case of a patient with nephrotic syndrome with a membranoproliferative glomerulonephritis pattern, with nodular glomerulosclerosis and predominance of histiocytes, including numerous lysosomes, which was initially thought to be crystal-storing histiocytosis (CSH) or light-chain deposition disease (LCDD). However, no crystalline structure was found in histiocytes in the glomeruli or interstitium in our patient. Moreover, no other causes of nondiabetic nodular sclerosis were identified (eg, smoking, Takayasu disease, cystic fibrosis, and cyanotic congenital heart disease).^4^

In 2016, Shah et al^5^ first reported a rare case of CSH, with histiocytes localized in the glomerular capillary loops but not in the interstitium. They described “crystals within macrophages (histiocytes) as recognized by numerous cytoplasmic vacuoles.” In 2019, Gupta et al^6^ also described 3 cases of CSH primarily affecting the glomerular capillaries. Polygonal crystalline inclusions were found in the cytoplasm of macrophages in 1 case, whereas “spherical/oval electron-dense crystals” were found in the other cases. However, it is difficult to morphologically distinguish between the “crystals within cytoplasmic vacuoles” described by Shah et al^5^ and the “spherical/oval crystals” described by Gupta et al^6^ from intracellular micro-organelles such as lysosomes and endosomes.

By definition, a “crystal” refers to a small piece of substance with several even sides, which is formed naturally when the substance becomes solid (Oxford Learner’s Dictionaries web). There have not been any pathologic definitions of “crystal,” although “crystal” generally represents rhomboidal, rectangular, or needle-shaped structures in crystal-associated diseases.

Our case indicated that the predominant histiocytic infiltration in the glomerular tuft was not crystalline in structure. In contrast, our findings revealed the presence of histiocytes containing numerous enlarged lysosomes that contained accumulated monoclonal free kappa light chains. Therefore, we diagnosed the patient in the present case with histiocytic glomerulopathy with noncrystalline inclusion associated with IgG-kappa plasma cell dyscrasia. This patient could be diagnosed with “non-crystalline histiocytosis.”

Stokes et al^7^ advocated for new classification of LCPT. Thus, they divided LCPT into 2 subtypes based on morphology, ie, crystalline and noncrystalline. We suspected that light chain–storing histiocytosis due to MGRS could also be classified into the crystalline and noncrystalline subtypes.

Herrera^2^ also divided LCPT into the following 4 groups based on characteristic immunomorphologic manifestations associated with specific clinical settings: (1) acute tubular injury or necrosis variant, (2) acute tubular damage with cytoplasmic inclusions, (3) acute tubulointerstitial nephritis type, and (4) lysosomal indigestion or constipation category. They suggested that the lysosomal indigestion or constipation variant was the least common subtype of LCPT. Because lysosomes cannot catabolize abnormal monotypical light chains, they proliferate, become larger, and acquire unusual shapes, leading to “indigestion and a constipated” appearance of proximal tubular cells. We assumed that a similar pathologic condition had developed in the histiocytes of glomeruli in the present case. Kaur and Sethi^8^ described a case of histiocytic glomerulopathy, secondary to macrophage-activating syndrome, following a viral infection. Although both the clinical course and pathogenesis may be different from those in our case, the histopathologic diagnosis, ie, histiocytic glomerulopathy, and the pathophysiology, ie, macrophage activation, could be in line with those in our case.

Notably, the pathologic findings in the present case closely and morphologically resemble LCDD using light microscopy. Considering the similarity, these mesangial nodular lesions could be formed because of pathologic monoclonal light chains via a similar pathophysiology in LCDD. Gokden et al^9^ described 46 cases of patients with LCDD; IF and EM results were available for 39 cases: 2 (6%) cases yielded negative results with IF but showed deposits with EM. Our case may be classified as LCDD with morphologic heterogeneity.^9^ We speculated that the negative findings in the GBM observed using IF and immunoelectron microscopy could have been due to aggressive phagocytosis by histiocytes.

Little information is available about the natural course, treatment, and prognosis of histiocytic glomerulopathy or CSH. Keane and Gill^10^ reported a case of CSH recurrence after kidney transplantation without treatment of underlying hematologic disorders. Watanabe et al^11^ reported a similar case due to IgG-lambda multiple myeloma successfully treated with BD therapy and autologous stem cell transplantation.

In conclusion, we reported a rare case of histiocytic glomerulopathy due to IgG-kappa. The importance of the nomenclature “histiocytic glomerulopathy with non-crystalline inclusion” is to reflect the association with these characteristic pathologic findings and underlying plasma cell dyscrasia so that the disease can be diagnosed as MGRS and treated appropriately.

## References

[1] Sethi S., Rajkumar S.V., D’Agati V.D. (2018). The complexity and heterogeneity of monoclonal immunoglobulin-associated renal diseases. J Am Soc Nephrol.

[2] Herrera G.A. (2014). Proximal tubulopathies associated with monoclonal light chains: the spectrum of clinicopathologic manifestations and molecular pathogenesis. Arch Pathol Lab Med.

[3] Larsen C.P., Messias N.C., Walker P.D. (2015). Membranoproliferative glomerulonephritis with masked monotypic immunoglobulin deposits. Kidney Int.

[4] Nasr S.H., D'Agati V.D. (2007). Nodular glomerulosclerosis in the nondiabetic smoker. J Am Soc Nephrol.

[5] Shah S., Sethi S., Arend L., Geetha D. (2016). Crystal-storing histiocytosis. Kidney Int.

[6] Gupta R.K., Rosenberg A.Z., Bagnasco S.M., Arend L.J. (2019). Renal crystal-storing histiocytosis involving glomeruli—a comprehensive clinicopathologic analysis. Ann Diagn Pathol.

[7] Stokes M.B., Valeri A.M., Herlitz L. (2016). Light chain proximal tubulopathy: clinical and pathologic characteristics in the modern treatment era. J Am Soc Nephrol.

[8] Kaur A., Sethi S. (2016). Histiocytic and nonhistiocytic glomerular lesions: foam cells and their mimickers. Am J Kidney Dis.

[9] Gokden N., Barlogie B., Liapis H. (2008). Morphologic heterogeneity of renal light-chain deposition disease. Ultrastruct Pathol.

[10] Keane C., Gill D. (2008). Multi-organ involvement with crystal-storing histiocytosis. Br J Haematol.

[11] Watanabe H., Osawa Y., Goto S. (2015). A case of endocapillary proliferative glomerulonephritis with macrophages phagocytosing monoclonal immunoglobulin lambda light chain. Pathol Int.

